# The impact of nasal valve surgery on snoring

**DOI:** 10.1016/j.bjorl.2026.101887

**Published:** 2026-10-02

**Authors:** Roberto Campos Meirelles, Miguel Soares Tepedino, Pedro Miño Vianna, João Pedro Resende Cantarini Oliveira

**Affiliations:** Universidade do Estado do Rio de Janeiro, Rio de Janeiro, RJ, Brazil

**Keywords:** Nasal obstruction, Snoring, Rhinoplasty, Sleep, Airway resistance

## Abstract

**Objectives:**

Snoring is a common complaint in ENT and sleep medicine clinics, with significant social relevance and potentially indicating more serious conditions, such as upper airway resistance syndrome and obstructive sleep apnea. This prospective analytical study evaluated the impact of nasal surgery on reducing snoring duration in patients with a history of nasal obstruction.

**Methods:**

Thirty patients were selected and divided into two groups: Group 1, consisting of patients with complex septal deviations, nasal valve weaknesses, visible nasal structural changes, and a history of snoring; and Group 2, with moderate to severe septal deviations and snoring complaints. The duration of snoring as a percentage of sleep time was measured using the SnoreLab™ mobile app. Additionally, nasal obstructive symptoms, risk of obstructive sleep apnea, excessive daytime sleepiness, and nasal aesthetic satisfaction were assessed using specific scales.

**Results:**

The results showed a significant reduction in snoring time in Group 1. A weak association was found between chronic nasal obstruction and increased risk of obstructive sleep apnea in both groups.

**Conclusion:**

We conclude that surgical correction of nasal obstructions may be an effective approach to reducing snoring, emphasizing the importance of a thorough assessment of nasal anatomy in patients with complaints of nasal obstruction and snoring.

**Level of evidence:**

The study, titled “The Impact of Nasal Valve Surgery on Snoring”, is classified as Level III evidence. This level is based on the study's design as a prospective analytical study without randomization. Observational prospective studies like this one, which track patients over time to evaluate outcomes without the use of randomized control groups, are typically categorized as Level III evidence according to commonly accepted evidence hierarchies in clinical research.

## Highlights

• Nasal surgery offers effective snoring mitigation.
• Nasal surgery offers effective snoring mitigation.
• Research advocates for detailed nasal anatomy assessment in snoring patients.
• Associative study highlights the role of nasal obstructions in sleep disorders.

## Introduction

Since 1973, when Guilleminault identified patients with respiratory effort-related micro-arousals without hypoxemia, the Upper Airway Resistance Syndrome (UARS) has been recognized as a pathologic entity.^1^^,^^2^ Associated with daytime drowsiness, snoring, and sleep problems, this syndrome has since become a focus of study.^3^ Snoring, a marker of both UARS and obstructive sleep apnea, varies according to age and sex and is critical for diagnosis of these conditions.^4^^,^^5^ Advances in research have provided a better understanding of nasal breathing and its role in sleep disorders.^6^

Recent studies have associated habitual snoring with an increased risk of cerebrovascular and cardiovascular events – such as stroke and Coronary Artery Disease (CAD), increasing the risk of CAD by 28% – and suggested a possible role in the accelerated formation of carotid plaque. These findings indicate that snoring, by impacting endothelial function and causing inflammation, is a modifiable risk factor for the aforementioned diseases.^7^^,^^8^

The need for accurate diagnosis has driven the use of polysomnography as the gold-standard method, but its high cost and limited availability have encouraged alternative technologies for sleep monitoring.^9^ Smartphone apps, despite limitations, have proven useful for tracking sleep-disordered breathing. Mobile apps that record sound overnight sleep have demonstrated impressive sensitivity for snore detection. High accuracy values – up to 95% – have been a recurring finding among recent studies on the subject.^10^^,^^11^

Within this context, the present article examines the evolution of nasal valve surgery and its impact on snoring, using smartphone-based monitoring technologies.

## Objectives

The primary objective of this study was to determine the impact of nasal surgical procedures on the percentage of time spent snoring over a single night’s sleep, by comparing the duration of snoring as measured by the SnoreLab™ (SL) (Reviva Softworks Ltd., London, UK) mobile application before and after surgery.

Additionally, the relationship between the severity of nasal obstruction and factors such as daytime drowsiness, snoring sleepiness, risk of sleep apnea, and perception of nasal aesthetics was examined using questionnaires.

## Methods

This article describes a prospective controlled study conducted at the outpatient ENT clinic of a tertiary university hospital between December 2020 and December 2022. Thirty patients with complaints of snoring and nasal obstruction refractory to medical treatment, who underwent surgery for chronic nasal obstruction, were included in the sample.

Participants were divided into two groups based on characteristics of septal deviation, nasal valve behavior, and lateral cartilage integrity. Group 1 included patients with complex or “difficult” septal deviations, involving caudal deflections in Cottle’s area 1 and marked irregularities in areas 2–4, frequently classified as grade III. These individuals also exhibited asymmetries and weakening of the upper and lower lateral cartilages. On physical examination, they presented with nasal tip ptosis, inspiratory collapse of the nasal valves due to ligament laxity, widened medial crural footplates, asymmetric nostrils, and deviated nasal pyramids. Group 2 comprised patients with moderate to severe septal deviations (grades II or III) confined to Cottle’s areas 2–4, all of which were exclusively intranasal and not visible without anterior rhinoscopy, endoscopy, or CT imaging.^12^, ^13^, ^14^

Individuals were excluded if they were under 18 or over 60-years of age, had craniofacial syndromes, adenoidal hypertrophy occupying more than 20% of the nasopharyngeal space, Brodsky grade III or IV palatine tonsils, or a diagnosis of chronic rhinosinusitis. Additionally, patients with a Body Mass Index (BMI) equal to or greater than 30 kg/m^2^, or with neck circumference exceeding 43 cm for men and 38 cm for women, were excluded. These cutoffs were based on established thresholds in the literature associated with increased upper airway collapsibility and elevated risk of obstructive sleep apnea. The application of these anthropometric criteria during screening helped to reduce confounding variables unrelated to nasal anatomy and ensure a more homogeneous sample.

The SL (SnoreLab™) smartphone application was used to objectively assess snoring time before and after surgery. All participants received a standardized institutional video with detailed instructions on proper use of the app, including ideal phone positioning (30–100 cm from the head, aligned at pillow height), preference for lateral decubitus, solitary sleeping conditions, and elimination of ambient noise sources such as pets or televisions. Each patient completed three full nights of recording at both the preoperative and postoperative stages. To minimize variability, the intermediate night was selected for analysis, while the first and third nights were excluded to avoid the effects of acclimatization or fatigue. Although various smartphones were used, inclusion required compatibility with the latest SL version and adequate microphone sensitivity, reducing potential variability related to hardware differences. The 3-month postoperative interval was selected to ensure complete mucosal healing, resolution of inflammation, and stabilization of nasal airflow dynamics, allowing for meaningful and physiologically consistent comparisons.

Participants also completed validated questionnaires to assess nasal obstruction (NOSE scale), nasal aesthetic satisfaction (ROE), and sleep-related symptoms, including daytime drowsiness (Epworth Sleepiness Scale), snoring impact (Stanford Snoring Scale), and obstructive sleep apnea risk (Berlin Questionnaire). All participants signed informed consent prior to enrollment.^15^, ^16^, ^17^, ^18^, ^19^, ^20^

### Surgical procedure

All procedures were performed by the same surgeon. Participants in Group 1 underwent open rhinoseptoplasty. For treatment of the nasal tip, the latero-lateral septal extension graft technique was adopted, with a septal cartilage graft placed contralateral to the deviation to centralize the septum and apply crural tensioning.^21^^,^^22^ Laterally deviated nasal pyramids were corrected by osteotomies and bilateral placement of spacer grafts, along with articulated alar rim grafting to support nasal valve function and contour.^23^^,^^24^

In Group 2, septoplasty was performed endoscopically using the modified Cottle technique.^25^^,^^26^

In both groups, all bony and cartilaginous deviations were corrected, and inferior turbinoplasty was performed using a standardized approach: lateral outfracture of the inferior turbinate, submucosal resection, and bipolar cauterization for hemostasis, with preservation of the mucosal surface.^27^^,^^28^ Nasal splints were placed in all patients and removed after 7-days to complete postoperative care.

Although the turbinoplasty technique was identical across both groups, the extent of structural intervention varied considerably. Group 1 procedures involved both internal and external nasal framework reconstruction, while Group 2 underwent intranasal correction only. This surgical heterogeneity may partially explain the differences observed in functional outcomes.

The single-surgeon protocol ensured procedural consistency, but also represents a potential source of operator bias, limiting external generalizability (Fig. 1).

**Fig. 1:**
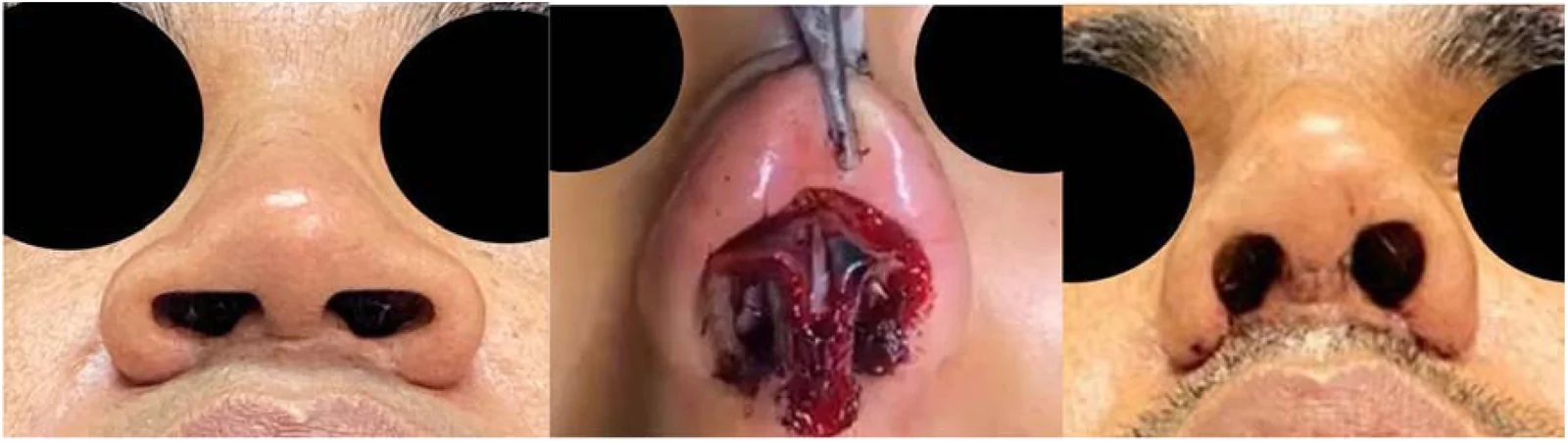
Preoperative (left), intraoperative (center), and postoperative (right) images of a representative patient from Group 1.

### Data analysis

For data analysis, two-factor repeated-measures analysis of variance (ANOVA) was used, evaluating the effects of time point and group on the primary outcomes, with Tukey’s tests to identify significant differences and Levene’s method to verify the homogeneity of variances.

For secondary outcomes, Fisher’s exact test for categorical variables in small samples was used, and the Shapiro-Wilk test to verify the assumption of normality. Depending on the normality of the data, one-way ANOVA or the Kruskal-Wallis test with Spearman correlation were applied, with significance established at α = 0.05. ANOVA was conducted in Jamovi software™ (Jamovi Project, Sydney, Australia), while all other analyses were performed in *R*.^29^^,^^30^

## Results

### Primary outcome

The effect of rhinoseptoplasty and septoplasty on snoring time was evaluated in 30 participants (15 per group) using the SnoreLab™ mobile application, with recordings obtained both pre- and postoperatively. A two-way repeated-measures ANOVA was performed to assess the influence of time and surgical group. Outliers were defined as values exceeding ±2 standard deviations from the respective group mean and were excluded to enhance data reliability, as they likely represented recording artifacts or technical inconsistencies. One outlier was excluded from each group, resulting in a final sample of 28 participants for statistical comparison.

Following reanalysis, the model demonstrated a significant main effect of time (p < 0.001), a group effect (p = 0.028), and a significant interaction between time and group (p = 0.013), as shown in Table 1. Post hoc analysis using Tukey’s test revealed that the rhinoseptoplasty group had significantly higher baseline snoring time compared to the septoplasty group (p = 0.047) and experienced a significant postoperative reduction (p < 0.001). In contrast, the septoplasty group showed a non-significant decrease in snoring time (p = 0.064). No significant difference was observed between the groups at the postoperative time point (p = 0.829).

**Table 1:** Two-way repeated-measures ANOVA and Tukey post hoc comparisons of SnoreLab™ snoring time (%) between rhinoseptoplasty (Group 1) and septoplasty (Group 2) groups, at preoperative and postoperative time points.

Factor	SS	df	MS	F	p
Time	1399	1	1398.6	39.11	<0.001
Time × Group	256	1	256.0	7.16	0.013
Residuals (within)	930	26	35.8		
Group	533	1	532.8	5.42	0.028
Residuals (between)	2554	26	98.2		

Comparison	MD	SE	df	p
Pre-Rhino vs. Pre-Septo	10.47	3.78	26	0.047
Pre-Rhino vs. Post-Rhino	14.31	2.35	26	<0.001
Pre-Rhino vs. Post-Septo	16.21	3.15	26	<0.001
Pre-Septo vs. Post-Rhino	3.84	3.05	26	0.596
Pre-Septo vs. Post-Septo	5.73	2.18	26	0.064
Post-Rhino vs. Post-Septo	1.90	2.23	26	0.829

SS, Sum of Squares; df, Degrees of freedom; MS, Mean Square; MD, Mean Difference; SE, Standard Error; Rhino, Rhinoseptoplasty; Septo, Septoplasty; Pre, Preoperative; Post, Postoperative.

According to the Shapiro-Wilk test, only preoperative SL snoring time values in the septoplasty group did not follow a normal distribution (p < 0.05). The Levene test confirmed the homogeneity of variances at the preoperative (*F* = 0.657; p = 0.425) and postoperative (*F* = 0.001; p = 0.970) time points, validating the necessary assumptions for ANOVA.

### Secondary outcome

There were no significant differences between the two groups in the baseline scores of the NOSE, ROE, Epworth, Stanford Snoring, or Berlin questionnaires (p > 0.05 for all comparisons, Fisher’s exact test). Postoperatively, both groups demonstrated improvement across all scales, although only the rhinoseptoplasty group showed statistically significant reductions in NOSE and Stanford Snoring scores (p < 0.05). Changes in ROE and Epworth scores followed similar trends but did not reach statistical significance.

To specifically evaluate whether subjective nasal obstruction was associated with lower snoring levels after surgery, a Spearman correlation analysis was conducted between NOSE scale scores and objective snoring time measured by the SnoreLab™ app. The results showed a weak and non-significant correlation both preoperatively (ρ = 0.19, p = 0.32) and postoperatively (ρ = 0.16, p = 0.41). Therefore, no significant linear association was found between self-reported nasal obstruction severity and objectively recorded snoring time, either before or after surgery. This suggests that subjective perception of obstruction alone is not a reliable predictor of snoring duration, reinforcing the multifactorial etiology of snoring and the importance of comprehensive anatomical assessment beyond patient-reported symptoms (Table 2).

**Table 2:** Distribution of patients across questionnaire score categories (NOSE, ESS, ROE, Stanford, Berlin) for Groups 1 (rhinoseptoplasty) and 2 (septoplasty), with p-values from Fisher’s exact test.

Group 1 ‒ Rhinoseptoplasty (n = 15)						
Scale	Category	Mild (n = 2)	Moderate (n = 2)	Severe (n = 5)	Extreme (n = 6)	p
ESS	Normal	1 (50)	0 (0)	0 (0)	2 (33.3)	0.23
	Moderate	1 (50)	1 (50)	1 (20)	0 (0)	
	Abnormal	0 (0)	1 (50)	4 (80)	4 (66.7)	
ROE	≤7	1 (50)	1 (50)	2 (40)	4 (66.7)	0.88
	>7	1 (50)	1 (50)	3 (60)	2 (33.3)	
Stanford	≤3	0 (0)	2 (100)	4 (80)	5 (83.3)	0.16
	>3	2 (100)	0 (0)	1 (20)	1 (16.7)	
Berlin	Low risk	1 (50)	1 (50)	0 (0)	2 (33.3)	0.33
	High risk	1 (50)	1 (50)	5 (100)	4 (66.7)	

Group 2 ‒ Septoplasty (n = 15)						
Scale	Category	Mild (n = 1)	Moderate (n = 4)	Severe (n = 7)	Extreme (n = 3)	p
ESS	Normal	0	1 (33.3)	1 (33.3)	0 (0)	1.00
	Moderate	0	0 (0)	0 (0)	1 (50)	
	Abnormal	0	2 (66.7)	2 (66.7)	1 (50)	
ROE	≤6	1 (100)	4 (100)	3 (42.9)	1 (33.3)	0.21
	>6	0 (0)	0 (0)	4 (57.1)	2 (66.7)	
Stanford	≤1	1 (100)	1 (25)	5 (71.4)	3 (100)	0.17
	>1	0 (0)	3 (75)	2 (28.6)	0 (0)	
Berlin	Low risk	0 (0)	3 (75)	2 (28.6)	2 (66.7)	0.39
	High risk	1 (100)	1 (25)	5 (71.4)	1 (33.3)	

Fig. 2, Fig. 3 use Spearman coefficients to illustrate the relationship between the NOSE scale and the ESS, ROE, and Stanford instruments, including linear regressions and confidence intervals. In the Rhinoseptoplasty group, a weak to moderate linear correlation was observed between NOSE scores and those on the other scales, without reaching statistical significance. In the Septoplasty group, correlations ranged from negligible to moderate and again were not significant.

**Fig. 2:**
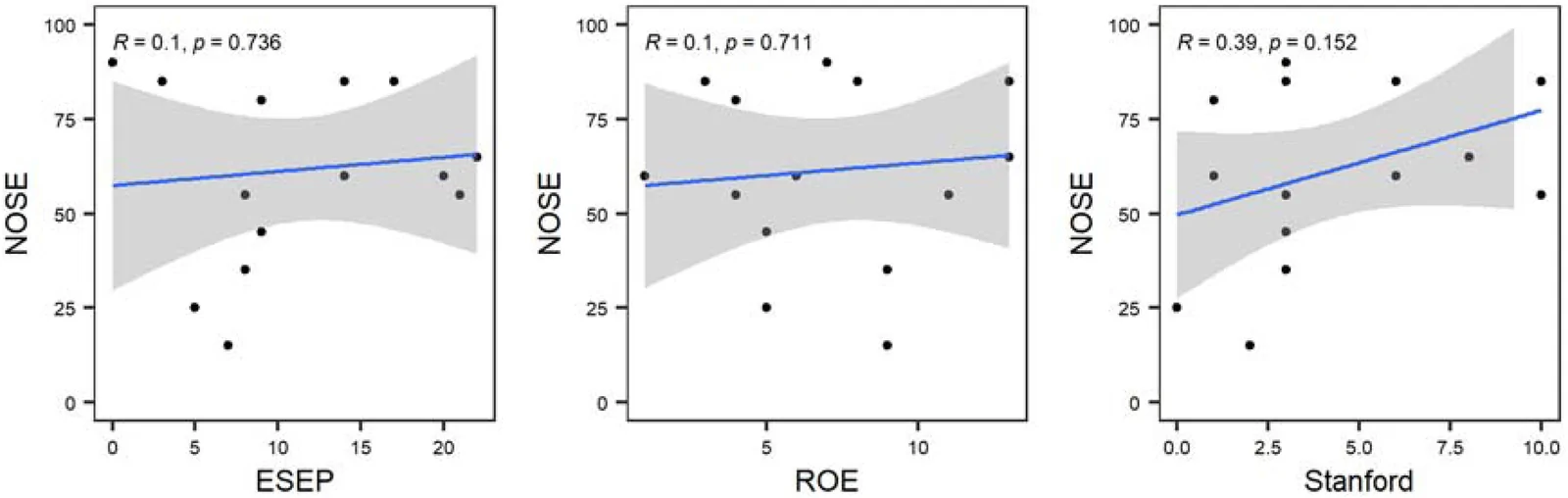
Analysis of correlation between scales, Rhinoseptoplasty group.

**Fig. 3:**
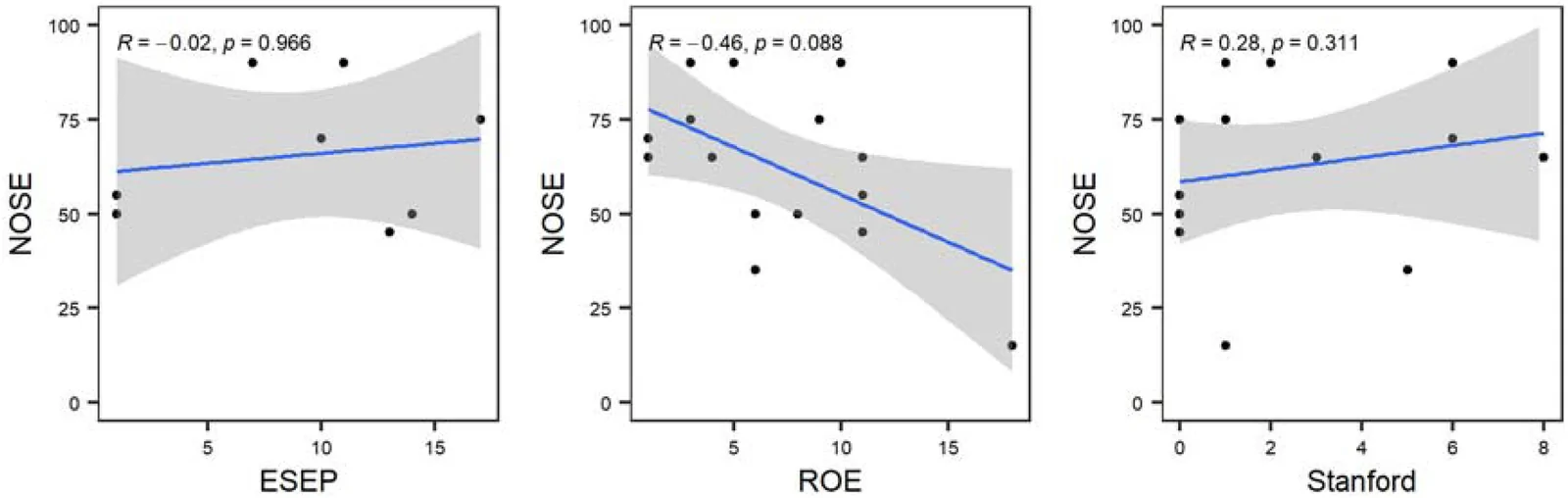
Analysis of correlation between scales, Septoplasty group. ESEP, Epworth Sleepiness Scale; ROE, Rhinoplasty Outcomes Evaluation.

Table 3 compares ESS, ROE, and Stanford scores by NOSE score categories in Groups 1 and 2, with means and standard deviations. ANOVA or the Kruskal-Wallis test (as appropriate) confirmed the absence of any significant differences between scale scores. Numerically, values did vary across NOSE categories, but the differences never reached statistical significance. These results suggest that, within each surgical group, the degree of subjective nasal obstruction (as categorized by the NOSE scale) was not significantly associated with daytime sleepiness, aesthetic satisfaction, or snoring impact as measured by the corresponding instruments.

**Table 3:** Mean scores (±SD) and medians of ESS, ROE, and Stanford questionnaires stratified by NOSE severity category for Groups 1 (rhinoseptoplasty) and 2 (septoplasty).

Group 1 ‒ Rhinoseptoplasty (n = 15)					
Scale	NOSE category	n	Mean (SD)	Median	p
ESS	Mild	2	6.00 (1.41)	6.00	0.11^a^
	Moderate	2	8.50 (0.71)	8.50	
	Severe	5	17.00 (5.92)	20.00	
	Extreme	6	9.50 (6.77)	11.50	
ROE	Mild	2	7.00 (2.83)	7.00	0.98^a^
	Moderate	2	7.00 (2.83)	7.00	
	Severe	5	7.00 (4.95)	6.00	
	Extreme	6	8.00 (4.29)	7.50	
Stanford	Mild	2	1.00 (1.41)	1.00	0.35^a^
	Moderate	2	3.00 (0.00)	3.00	
	Severe	5	5.60 (3.65)	6.00	
	Extreme	6	4.83 (3.19)	4.50	

Group 2 ‒ Septoplasty (n = 15)					
Scale	NOSE category	n	Mean (SD)	Median	p
ESS	Moderate	1	7.00	7.00	0.94^a^
	Severe	3	9.33 (8.02)	10.00	
	Extreme	4	9.75 (5.97)	12.00	
ROE	Mild	2	14.50 (4.95)	14.50	0.056^a^
	Moderate	2	7.00 (2.83)	7.00	
	Severe	5	4.40 (2.70)	4.00	
	Extreme	6	7.00 (4.34)	8.00	
Stanford	Mild	2	2.00 (1.41)	2.00	0.98^b^
	Moderate	2	1.50 (0.71)	1.50	
	Severe	5	2.80 (2.77)	3.00	
	Extreme	6	2.50 (3.56)	0.50	

a ANOVA.

b Kruskal-Wallis.

The results show that patients in Group 1 experienced longer-lasting snoring and worse scores on the study scales compared to Group 2, suggesting that conditions such as septal deviation and collapsible nasal valves contribute to more severe nasal obstruction symptoms, which in turn lead to deterioration in sleep quality. Regarding the primary outcome, only Group 1 showed a statistically significant reduction in percent time spent snoring, although a decrease in snoring was noted in both groups.

Although the secondary objectives did not reach statistical significance, there was a weak to moderate correlation between scores on the NOSE scale and those of the other questionnaires in Group 1 and a slight correlation with the ESS and Stanford scales in Group 2, as well as a negative correlation with the ROE scale.

## Discussion

Starting from the premise that the nasal cavity accounts for approximately half of airway resistance, our study investigated whether surgical correction of narrow nasal passages could impact airflow dynamics and soft-tissue vibration, as per Bernoulli’s principle.^31^, ^32^, ^33^ Our hypothesis was that such interventions would reduce snoring and respiratory effort. Our data confirmed this hypothesis: patients in Group 1, who had more severe restriction of nasal passages, had worse preoperative snoring and were the only group to show a significant reduction in the percent time spent snoring during sleep.

Studies have shown that facilitating nasal breathing can provide several benefits, including reduction of oral breathing (which can contribute to the narrowing of the oropharynx due to changes in the position of the base of the tongue and jaw) and improvement of nitric oxide transport through the airway, reducing episodes of sleep apnea.^34^^,^^35^ Although the secondary objectives of the study did not reach statistical significance, a weak and non-significant Spearman correlation was found between the NOSE scale (a measure of subjective nasal obstruction) and objective snoring time measured by the SL app, both preoperatively (ρ = 0.19, p = 0.32) and postoperatively (ρ = 0.16, p = 0.41). In other words, patients who reported greater improvement in subjective nasal obstruction did not necessarily exhibit proportionally lower snoring times. This finding reinforces the multifactorial nature of snoring and highlights that nasal obstruction is one contributing factor, but not the sole determinant of sleep-disordered breathing severity. It also underscores the need for objective measures ‒ such as rhinomanometry or polysomnography ‒ to complement patient-reported outcomes in future investigations.

Anderson’s nasal tripod and Mink’s internal nasal valve are two classic anatomical concepts or theories. Anderson describes the nasal structure as a tripod formed by the crura of the alar cartilage, which would be essential for preserving aesthetics and functionality. Mink, in turn, identified the internal nasal valve (formed medially by the superior portion of the nasal septum, superiorly and laterally by the caudal portion of the upper lateral cartilage, and inferiorly by the head of the inferior turbinate) as the narrowest region of the nasal cavity, and thus crucial for efficient airflow.^36^^,^^37^

The present study highlights the diversity of nasal abnormalities and the risk of underestimating improvement in symptoms, such as snoring, when generalizing patients’ nasal features. Our findings reinforce the three-dimensional approach proposed by Howard D. Stupak, which divides the nose into layers: foundation (facial and septal bones), framework (septum and nasal bones), and canopy (alar cartilages and skin). Described as a triangular prism, Stupak’s model promotes a balanced, integrative approach to the nose, focusing on precise alignment of the nasal tip, correction of the septum, and optimal positioning of the alar cartilages. This perspective allows for a detailed, individualized analysis of surgical needs, laying a solid groundwork for precise and personalized interventions.^38^

The results of the present study corroborate this approach, by demonstrating that patients with more complex septal deviations respond differently to surgery compared to those with less severe deviations, thus highlighting the need for a detailed, specific, case-by-case assessment of nasal anatomy. Furthermore, the study proposes the possibility of, in future, stratifying nasal valve dysfunction in a manner analogous to the Friedman criteria, which are applied to recommend pharyngeal surgical procedures in the treatment of sleep apnea.^39^ This stratification would not only allow the surgeon to predict the success rates of nasal surgery in improving snoring with greater accuracy, but also highlight that careful observation of details to be corrected could exponentially enhance the operative outcome. This is borne out by Poiseuille’s law, which postulates that a 10% increase in nasal cross-sectional area results in a 21% increase in airflow, thus reducing the Bernoulli effect, which is responsible for mucosal vibration and, consequently, snoring.^40^ However, without direct physiologic measures such as rhinomanometry or PSG-derived airflow metrics, it is not possible to confirm whether the observed reduction in snoring time directly results from changes in airflow dynamics or other confounding variables.

Adopting such an approach would not only refine precision in surgical indications, but also improve our understanding of the potential impact of nasal interventions, allowing for more focused, effective treatments for sleep-related breathing disorders. It is important to note that all patients underwent inferior turbinoplasty, a factor that may have contributed to improvements in nasal airflow independently of the valve or septal correction. Although the surgical technique was standardized and applied symmetrically across both groups, isolating the specific contribution of nasal valve surgery remains challenging. This overlap represents a limitation of the present design, which should be addressed in future studies through additional control groups or stratified analyses.

Several limitations must be acknowledged. First, although the SnoreLab™ mobile application enabled the collection of multiple nights of data under real-world conditions, it is not equivalent to polysomnography and cannot detect apneas, hypopneas, or oxygen desaturation events. While prior studies suggest moderate agreement between SnoreLab and PSG in estimating snoring time, the app is limited to audio-based tracking and cannot replace the comprehensive diagnostic scope of PSG. Second, despite patients following standardized instructions ‒ including sleeping alone, keeping the smartphone 30–100 cm from the head, and recording under quiet conditions with the aid of a guided video ‒ only the intermediate night of three was analyzed, which may not fully eliminate night-to-night variability. Third, all patients underwent inferior turbinoplasty in addition to their primary surgical procedure. While the technique was standardized, its contribution to improved airflow could not be independently quantified, potentially confounding the specific impact of nasal valve correction. Fourth, the study was not randomized and lacked a non-surgical or sham control group, limiting causal inference and leaving room for placebo effects or spontaneous improvement. In addition, all procedures were performed by a single surgeon, ensuring technical consistency but introducing potential operator bias. The three-month follow-up period does not allow for conclusions regarding the long-term durability of surgical outcomes. Lastly, the small sample size and absence of a pre-specified power calculation reduce the robustness of subgroup analyses and increase the risk of Type II error, particularly in comparisons where no statistically significant differences were observed.

Still, it is worth noting that the SnoreLab™ app was well accepted by participants, suggesting its potential utility in future research settings ‒ provided its limitations are clearly acknowledged and it is not used as a substitute for formal polysomnography.

Traditionally, the term “deviation” has been used to describe a simple linear displacement; however, the surgical reality is far more complex. Deviated septa often present with complex deformations and bends, defying anatomical expectations and complicating surgical procedures. These are often compounded by structural changes in the nasal pyramid and lateral cartilages, which require an in-depth understanding and adaptation of surgical approaches to each specific case.

Although nasal surgery is commonly seen as essential for treatment of sleep-disordered breathing, research exploring this relationship continues to show mixed results. Advances in our understanding of nasal anatomy and physiology are crucial to improving knowledge of these pathological conditions. This study, in particular, suggests that surgical procedures can have a positive impact on snoring and sleep quality, highlighting the importance of supporting the nasal valves and correcting any septal deviations for treatment to be effective.^41^, ^42^, ^43^, ^44^

It should be emphasized that the present study did not include Polysomnography (PSG) or any objective diagnostic tool for Obstructive Sleep Apnea (OSA). As a result, it was not possible to determine whether the observed snoring reductions were accompanied by changes in Apnea-Hypopnea Index (AHI), oxygen desaturation levels, or sleep architecture. This is a relevant limitation, as snoring and OSA share common pathophysiological mechanisms but are not interchangeable endpoints. Incorporating PSG or validated home sleep testing devices in future studies would strengthen the clinical relevance of the findings by enabling a comprehensive evaluation of the impact of nasal valve surgery on both snoring and obstructive respiratory events during sleep. Such an approach would also help clarify whether the improvements in snoring time observed in Group 1 translate into meaningful reductions in sleep apnea severity.

## Conclusion

In this prospective study, patients with complex nasal anatomical alterations ‒ particularly those involving nasal valve dysfunction ‒ experienced significant reductions in snoring time following rhinoseptoplasty. In contrast, patients with isolated septal deviations who underwent endoscopic septoplasty did not exhibit statistically significant improvement. These findings highlight the importance of individualized anatomical analysis in the surgical management of sleep-disordered breathing symptoms such as snoring.

While the use of mobile applications such as SnoreLab™ offers practical benefits and was well accepted by participants, it should be viewed as a complementary tool rather than a substitute for gold-standard assessments like polysomnography. The study’s limitations ‒ including small sample size, short follow-up, absence of randomization and control group, and use of a single surgeon ‒ suggest that these results should be interpreted with caution.

Future research with larger cohorts, extended follow-up, and incorporation of objective airflow measurements (e.g., rhinomanometry or acoustic rhinometry) will be essential to confirm and expand upon these findings. Stratification based on nasal valve function may also aid in refining surgical indications and predicting outcomes more accurately.

## Funding

This research was self-funded and utilized the facilities provided by the tertiary hospital where the study was conducted.

## Declaration of competing interest

The authors declare no conflicts of interest.
